# Trends in paediatric inpatient antibiotic therapy in a secondary care setting

**DOI:** 10.1007/s00431-018-3185-z

**Published:** 2018-06-08

**Authors:** C. H. Quaak, E. Cové, G. J. Driessen, G. A. Tramper-Stranders

**Affiliations:** 1 Erasmus University, Rotterdam, the Netherlands; 2 ErasmusMC-Sophia Children’s Hospital, Rotterdam and Haga teaching hospital-Juliana Children’s Hospital, The Hague, the Netherlands; 3 Department of Pediatrics, Franciscus Gasthuis, Kleiweg 500, 3045 PM Rotterdam, the Netherlands; 4 Department of Neonatology, ErasmusMC-Sophia Children’s Hospital, Rotterdam, the Netherlands

**Keywords:** Antibiotic stewardship, Antimicrobial therapy, Secondary care hospital, Third-generation cephalosporins

## Abstract

There is growing attention for antimicrobial stewardship in paediatrics. Currently, little is known about secondary care antibiotic practice. We analysed trends in time with respect to inpatient antibiotic use in a secondary paediatric care setting. Total inpatient antibiotic consumption per year (2010–2015) and antibiotic prescriptions for urinary tract infection (UTI) and lower respiratory tract infection (LRTI) were analysed. Variables were total, antibiotic-specific, and intravenous days of therapy (DOT/100PD) and for UTI/LRTI treatment type, route and duration. Third-generation cephalosporin use decreased (DOT/100PD 11.6 in 2011 vs. 5.1 in 2015; *p* < 0.001); intravenous antibiotics were prescribed less often (*p* = 0.06). These findings were confirmed for the specific diseases: third-generation cephalosporin use decreased for both UTI (93% vs. 45%; *p* = 0.002) and LRTI (14% vs. 6%; *p* = 0.18); the duration of intravenous therapy decreased (UTI *p* = 0.02; LRTI *p* < 0.001). Median LRTI treatment duration was 9.2 days in 2008 and 6.6 in 2015 (p < 0.001); penicillin prescriptions were more narrow in spectrum (p = 0.02).

*Conclusion*: A decrease in third-generation cephalosporin use and intravenous route was identified. LRTI treatment was significantly shorter and more narrow in spectrum. This could be explained by awareness and interventions in the context of antimicrobial stewardship. A decrease in antibiotic use is also feasible and important in non-tertiary paediatric wards.

**Table:** 

**What is Known:** *• Antimicrobial stewardship programmes are effective in reduction of total and broad-spectrum antibiotic use in tertiary paediatric hospitals* *• The majority of hospitalised paediatric patients are admitted at general, secondary care wards, often for infectious diseases*	
**What is New:** *• Antimicrobial stewardship interventions in secondary care are also effective in establishing a reduction in broad-spectrum antibiotic use, intravenous route and days on antibiotic therapy*

**Electronic supplementary material:**

The online version of this article (10.1007/s00431-018-3185-z) contains supplementary material, which is available to authorized users.

## Introduction

Given the emergence of antimicrobial resistance and the impact on morbidity and mortality rates, as well as increased health care costs, appropriate antimicrobial use has become extremely important [11]. Antimicrobial stewardship refers to coordinated interventions designed to improve and measure the appropriate use of antimicrobials by promoting the selection of the optimal antimicrobial drug regimen, dose, duration of therapy, and route of administration. Antimicrobial stewards seek to achieve optimal clinical outcomes related to antimicrobial use, minimise toxicity and other adverse events, reduce the costs of health care for infections, and limit the selection for antimicrobial-resistant strains [1]. It also involves transparent monitoring of prescription data and use of available expertise and resources [12]. These elements could be accomplished by education, implementation of clinical practice guidelines, antimicrobial order forms, and dose optimization [4, 5]. Many studies have shown antimicrobial stewardship programmes (ASP) to be effective in reducing antibiotic prescribing, without negatively affecting the quality of care and patient mortality [19, 25].

ASP are also important in paediatrics. Children and neonates receive antibiotics frequently, because of the nature of childhood diseases [2, 13, 21]. The type of antibiotic prescriptions varies considerably between paediatric wards, despite similar clinical outcomes [10, 21]. Evaluations of paediatric ASP are limited compared to the number of evaluations of adult programmes [22]. Previous research has shown that ASP in hospitals are effective in reducing inappropriate antibiotic use in hospitalised children [16]. Total and specific broad-spectrum antibiotic use has decreased significantly in several paediatric hospitals in the USA since the introduction of the IDSA guidelines for ASP in 2007 [10, 17]. These declines were greater in hospitals with ASP than in hospitals without ASP. By routine monitoring, fewer antimicrobial medication errors were made [6, 10]. Medication prescription errors in paediatric patients are mainly made with antibiotics [7]. Moreover, for many infections, intravenous-to-oral switches could occur earlier [15].

There has been limited development of ASP in paediatrics, due to the lack of a standardised method of comparing antimicrobial use between hospitals [21]. Days of therapy (DOT) and defined daily dose (DDD) are recognised by the WHO to quantify antimicrobial use [9]. However, DDD does not take into account the weight and the age of the child. Therefore, DDD is a questionable paediatric methodology and the DOT is therefore preferable [21].

Currently, little is known about paediatric antibiotic use in the Netherlands and official ASP in paediatrics have not been introduced yet. Compared to other European countries, the numbers of antimicrobial prescriptions are low, but the number of inappropriate prescriptions is still worrying and antibiotic resistance is rising steadily [13]. Patterns of antibiotic prescription for specific diseases differ from country to country but also from one hospital to another [21]. Awareness of antibiotic prescription patterns could aid in identifying areas of inappropriate antibiotic prescribing and could contribute to a better antibiotic prescription policy. The aim of this study is to analyse qualitative and quantitative trends in time with respect to total and disease-specific antimicrobial agent use in a secondary paediatric care setting.

## Materials and methods

### Setting and ethics

This study was performed in the paediatric and neonatal ward of a secondary care urban teaching hospital in Rotterdam, the Netherlands (Franciscus Gasthuis, 21 paediatric beds; 18 neonatology beds including post-intensive care/high care). About 55% of the admissions on the neonatology wards are neonates born < 37 weeks of gestational age, but all are > 30 weeks of postmenstrual age. The most common infectious syndromes on the paediatric ward that require antibiotics are fever of unknown origin, lower respiratory tract infection, and urinary tract infection. In this hospital; since 2014, two consultant paediatricians regularly discuss antibiotic management and local guidelines with both the senior and junior doctors prior and after initiation of antimicrobial therapy. Moreover, in 2014, new protocols on lower respiratory and urinary tract infection were introduced. Two specific topics were studied: the total inpatient antibiotic use per year (2010 to 2015) and antibiotic prescriptions for two important infectious discharge diagnoses on the paediatric ward, namely lower respiratory tract infection (LRTI) and urinary tract infection (UTI) (2008 and 2015). The local ethics review board deemed formal ethical approval of the study unnecessary because of the absence of risk for patients. The data were stored anonymously.

### Data collection

Admission data for the paediatric and neonatology wards were obtained from the hospital database. Admission data from before 2010 could unfortunately not be reliably obtained. Single day admissions for diagnostic tests, chemotherapy courses, or blood transfusions were excluded. Children admitted for a non-paediatric speciality (e.g. surgery or ENT) were excluded because the paediatricians were not the primary responsible doctors. Since 2008, the pharmacy has stored all prescription data electronically. All prescriptions with the Anatomical Therapeutic Chemical classification-code (ATC) J01 for antibacterials were extracted from its computer system.

For the first part of the study, both databases were matched and the patient medical file was manually checked in case of differences in admission and antibiotic therapy duration. All files of children on co-trimoxazole (TMP/SMX) and trimethoprim therapy were checked manually; prophylactic prescriptions were excluded. Paediatric oncology patients (mainly leukaemia) were excluded from the analyses because in 2013, oncology treatment was discontinued in this hospital because of local policies. For the second part of the study, the pharmacy database was used to identify the children who were prescribed antibiotics for a LRTI or UTI in both 2008 (the first year data available) and 2015. Data were then retrieved from the medical files of these children.

### Variables

For the estimation of the total inpatient antibiotic use, variables were obtained per year and included age at admission, total number of admission days per year, days of antibiotic therapy (DOT; total, per ATC group, intravenous, and oral). The DOT per patient was calculated as the sum of antibiotics used per patient per day. Antibiotic consumption for the specific wards was expressed as DOT per 100 (in) patient-days (DOT/100PD) and estimated separately for every year. For the second part of the study, the following variables were noted: patient age, diagnostic parameters (C-reactive protein, white blood cell count, chest X-rays, cultures), comorbidity, admission duration, antibiotic prescriptions (including route of administration, dose, length of therapy, empirical and targeted/narrowed use). The total antibiotic prescription was studied, including discharge medication.

### Data analysis

Data were stored in an online research database (Castor EDC). Statistical analysis was performed with SPSS 23 (IBM SPSS Statistics).

Demographical data were analysed by descriptive statistics, including median and mean values with interquartile range or standard deviation for numerical data and proportions for categorical data. The Shapiro-Wilk test was used to test for normality of the distribution. Trends in DOT/100PD (total, per ATC group, oral, intravenous) for the period of 2010–2015 were analysed by time series analysis (ARIMA) for non-independent data. The data of the 2008/2015 patient cohorts were compared by using the Mann-Whitney *U* test, the chi-square, or Fisher’s exact test. *p* values < 0.05 were considered statistically significant.

## Results

### Trends in days of antibiotic therapy

The number of admissions and total admission duration per year are shown in Table 1. Additionally, details with respect to diagnoses are shown to compare case mix and severity during the years. There were no significant trends in patient case mix and severity; the admission duration seemed lower in 2015 compared to the other years and can be partly explained by the small percentage of early pre-terms (< 32 weeks of gestational age) admitted in 2015.

**Table 1:** Admission data

	2010	2011	2012	2013	2014	2015
Total admissions neonatology	382	423	460	390	392	410
Mean admission duration neonatology (days)	14.9	14.0	14.8	16.4	16.0	11.6
Total admission duration (days) neonatology	5675	5909	6783	6394	6220	4756
Percentage gestational age < 32 weeks	9.9	11.8	11.1	13.8	18.3	8.0
Total admissions paediatrics	1155	1218	1319	1256	1444	1465
Mean admission duration paediatrics (days)	4.5	3.9	3.8	3.4	3.4	3.2
Total admission duration (days) paediatrics	5209	4729	4897	4407	4636	4353
Percentage infectious diseases paediatrics						
Total	29.9	24.3	28.5	28.9	26.1	23.2
LRTI	9.1	8.8	9.8	9.4	8.6	8.4

### Neonatology ward

**Fig. 1:**
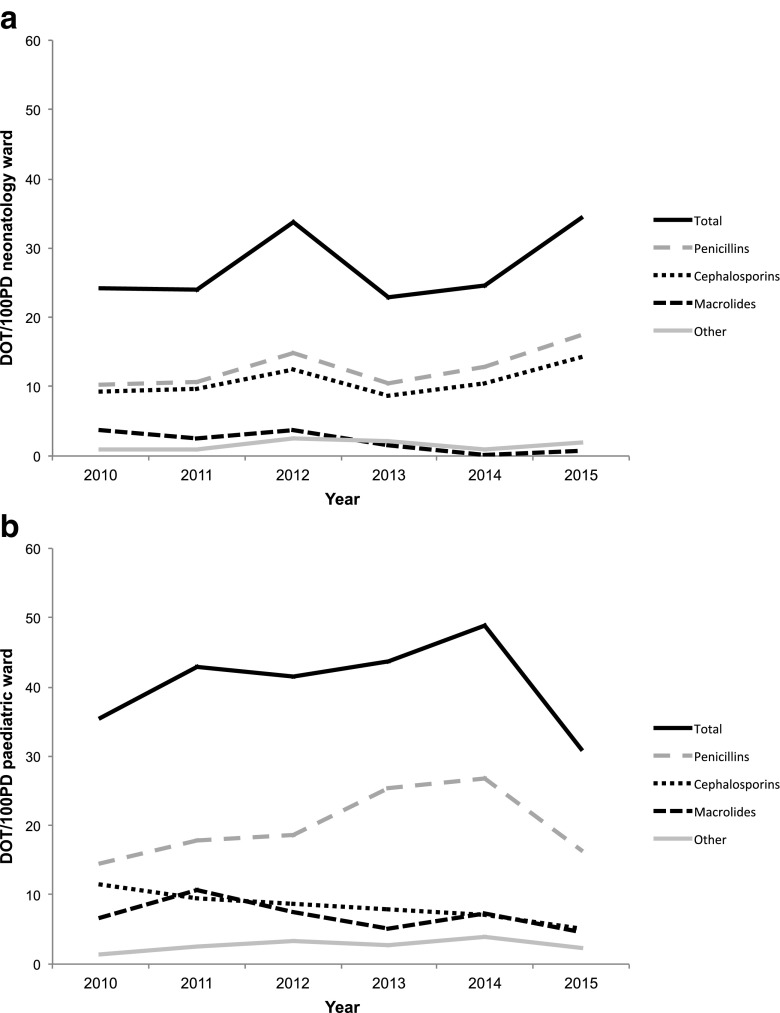
**a** Total DOT/100PD neonatology ward. DOT *R*^2^ = 0.17 (*p* = 0.42). ATC groups are shown in different shades. **b** Total DOT/100PD paediatric ward. Total DOT *R*^2^ = 2.7E−6 (*p* = 0.99). ATC groups are shown in different shades

The total DOT/100PD varied over the years from 22.9 to 34.4 (Fig. 1a). The most commonly prescribed antibiotics were beta-lactams, namely penicillins (J01C) and cephalosporins (J01D). The total DOT/100PD did not change in time (*R*^2^ = 0.17, *p* = 0.42). The combined penicillin and cephalosporin DOT/100PD increased from 20 to 32 but no significant differences in the DOT/100PD were calculated because of strong fluctuations. Antibiotics were mainly given intravenously (Fig. 1a, supplementary materials). No significant differences were seen in the route of administration of antibiotics over the years (*intravenous R*^2^ = 0.40, *p* = 0.18).

The post-delivery infants with risk factors for complications were observed in the maternity ward for several hours or even days, depending on the condition. The proportion of these infants who were prescribed antibiotics for suspected early-onset sepsis ranged from 2.1 to 7.5% per year; no trend in time was observed. For the majority of these infants (65.2–80.5%), antibiotic therapy was discontinued within 48 h because of negative blood culture, re-assuring clinical condition and inflammatory parameters.

### Paediatric ward

The total DOT/100PD varied over the years from 28.6 to 44.9 (Fig. 1b). No trend in time was observed (*R*^2^ = 2.7E−6, *p* = 0.99), although 2015 seems to show a decrease in DOT/100PD compared to the previous years. The beta-lactam penicillins (J01C) and cephalosporins (J01D) represent 73–84% of the total yearly DOT. Most frequently prescribed penicillins were amoxicillin and amoxicillin/clavulanic acid; no significant differences in the DOT were seen in this group of antibiotics over the years (*R*^2^ = 0.22, *p* = 0.36). Most frequently prescribed cephalosporins were cefotaxime and ceftriaxone. Cephalosporin prescriptions showed a significant decrease (*DOT/100PD 11.61 in 2011* vs. *5.10 in 2015*; *R*^2^ = 0.97, *p* < 0.001). The macrolide/lincosamide (J01F) group is the third most often prescribed group of antibiotics. No decrease was observed for this group (*R*^2^ = 0.32, *p* = 0.24).

The intravenous DOT/100PD varied from 9.9 to 20.5 and the oral DOT/100PD varied from 13.0 to 26.8 per year (Fig. 1b, supplementary materials). A trend was observed with respect to fewer intravenous DOT/100PD over the years (*R*^2^ = 0.63, *p* = 0.06).

### Top 3 inpatient antibiotic prescriptions per year

For every year, the top 3 of most often prescribed antibiotics on the paediatric ward was established. In 2010, ceftriaxone had a number 1 position, but through the years, ceftriaxone was replaced by amoxicillin and amoxicillin/clavulanic acid (place 1 and 2 respectively).

### Trends in total antibiotic prescriptions for urinary tract infections and respiratory tract infections

All patients admitted in 2008 and 2015 with a UTI or a LRTI were reviewed. The latter group consisted of patients with the discharge codes of pneumonia or bronchiolitis.

### Urinary tract infection

The baseline and microbiological characteristics of the patients included for urinary tract infection are shown in supplementary Table 1. Only gender was significantly different between the years. *E. coli* resistance for penicillins, cephalosporins, and TMP/SMX occurred more often in 2010 compared to 2015.

In Table 2, a comparison of the duration of antibiotic therapy between 2008 and 2015 is shown. The median total length of stay was 3 days in both years and the median total duration of treatment was 10 days in both years. The median total duration of intravenous therapy significantly decreased from 3 days in 2008 to 2 days in 2015 (*p* = 0.02).

**Table 2:** Characteristics of antibiotic therapy in patients with urinary tract infection

Characteristics	Year	*N*	Median (IQR)	Mean (SD)	*p* value*
Length of stay (days)	2008 2015	27 11	3.0 (3.0–5.0) 3.0 (2.0–5.0)	3.7 (1.6) 3.1 (1.9)	0.16
Total duration of treatment (days)	2008 2015	27 11	10.0 (6.0–10.0 10.0 (10.0–10.0)	8.0 (3.2) 9.7 (0.9)	0.22
Total duration of intravenous treatment (days)	2008 2015	27 11	3.0 (2.0–4.0) 2.0 (0.0–3.0)	3.1 (1.5) 1.8 (1.7)	0.02
Total duration of empirical treatment (days)^#^	2008 2015	27 11	3.0 (3.0–4.0) 3.0 (2.0–3.0)	3.7 (2.3) 2.7 (1.3)	0.23

*Mann-Whitney *U* test

^#^Duration until change of treatment according to culture results

**Fig. 2:**
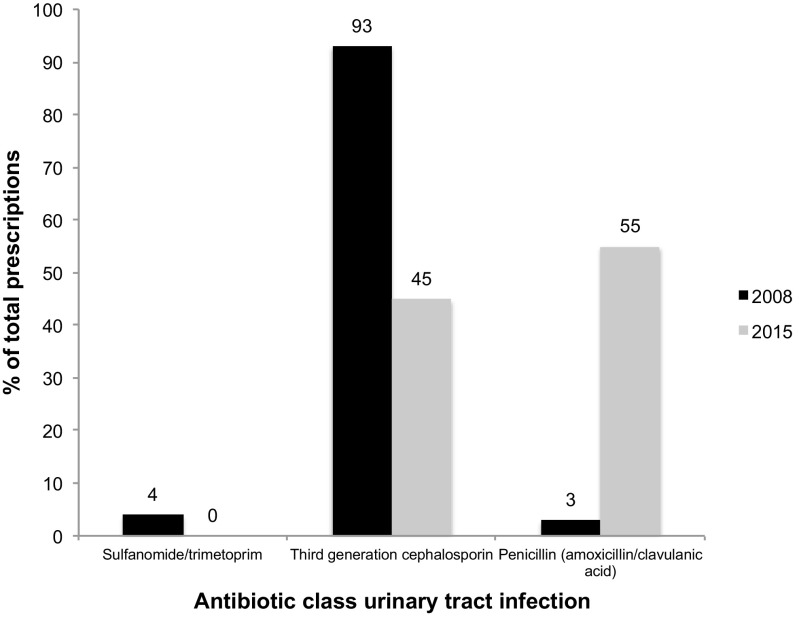
Empiric antibiotic therapy for urinary tract infection in 2008 and 2015. Cephalosporin prescriptions decrease significantly (chi-square, *p* < 0.01)

The empiric antibiotic therapy for both years is shown in Fig. 2. In 2008, cephalosporin antibiotics (93%) were the most commonly prescribed empirical antibiotics. In 2015, the number of empiric cephalosporin prescriptions was significantly lower (*93* vs. *45%*, *p* = 0.002) and replaced by penicillin antibiotics (amoxicillin/clavulanic acid). De-escalation and oral switch therapy were performed in 87 and 88% of patients in 2008, for 100% of patients in 2015 when deemed appropriate.

### Lower respiratory tract infection

The demographic and clinical characteristics of the patients included for LRTI are shown in supplementary Table 2. When the patients were admitted, they were all having low oxygen saturation (≤ 92% in room air) and/or apneas. In 2008, 262 patients were admitted for LRTI. Of these patients, 73 (27.8%) received antibiotic treatment. In 2015, 334 patients were admitted for LRTI. In total, 97 patients (29.0%) received antibiotic treatment.

The 2008 cohort had higher median inflammatory parameters; however, the number of patients tested was significantly lower in 2015. The number of additional investigations (blood cultures, chest X-rays, C-reactive protein, and white blood cell count) for LRTI decreased significantly over the years. Despite the decrease in additional investigations for LRTI, no difference was seen in the percentage of patients that started on antibiotics for LRTI.

In Table 3, characteristics of empiric antibiotic therapy are shown. The median length of stay in the hospital was for 3 days for both years. The total treatment duration decreased significantly (*p* < 0.001). A significant decrease was also noticed in total duration of intravenous treatment (*p* < 0.001). Intravenous-to-oral switch was performed for 96% of patients in 2008 and 100% in 2015 when deemed appropriate. In 2015, the majority of patients initiated with oral antibiotics (91.8 vs. 64.4% in 2008).

**Table 3:** Factors related to antibiotic therapy in patients with lower respiratory tract infection

Characteristics (unit)	Year	*N*	Median (IQR)	Mean (SD)	*p* value*
Length of stay (days)	2008 2015	73 97	3.0 (2.0–6.0) 3.0 (2.0–5.5)	4.5 (3.3) 4.3 (3.3)	0.69
Total duration of treatment (days)	2008 2015	73 97	7.0 (7.0–12.0) 7.0 (5.0–7.0)	9.2 (3.3) 6.6 (2.3)	< 0.01
Total duration of intravenous treatment (days)	2008 2015	73 97	0.0 (0.0–2.5) 0.0 (0.0–0.0)	1.4 (2.1) 0.3 (1.0)	< 0.01
Total duration of empirical treatment (days)	2008 2015	73 97	7.0 (7.0–12.0) 7.0 (5.0–7.0)	9.2 (3.3) 6.5 (2.3)	< 0.01

*Mann-Whitney *U* test

**Fig. 3:**
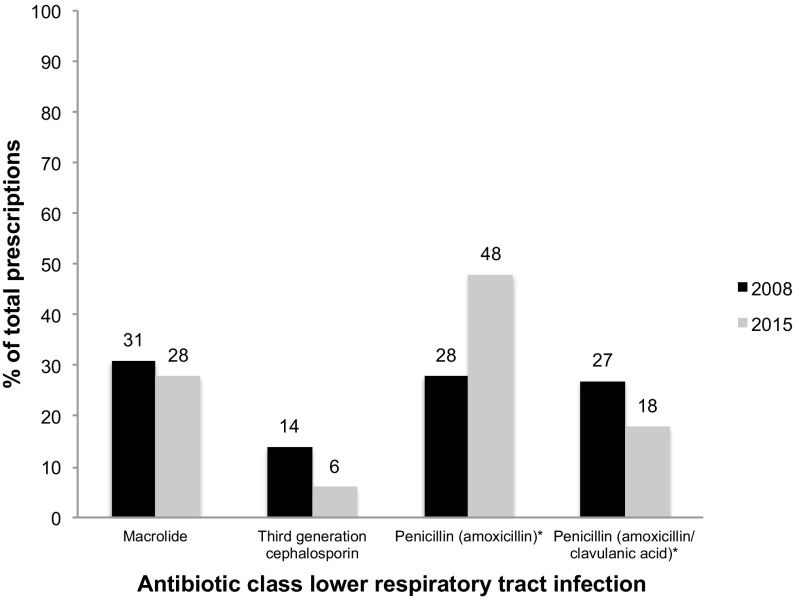
Empiric antibiotic therapy for lower respiratory tract infection 2008 and 2015. Cephalosporin prescriptions did not decrease significantly (chi-square, *p* = 0.18); a difference in penicillin prescriptions was observed (increase in amoxicillin and decrease in amoxicillin/clavulanic acid prescriptions in 2015; *p* = 0.02)

Figure 3 illustrates the empirical antibiotics for lower respiratory tract infection. For both years, penicillin antibiotics were the most commonly prescribed antibiotics (respectively 55 and 66%). The differences in penicillin, macrolide, and cephalosporin prescriptions were not significant (*p* = 0.18). However, the penicillin prescriptions were significantly more often smaller in spectrum in 2015 (*51% amoxicillin and 49*% *amoxicillin/clavulanic acid in 2008* vs. *72% amoxicillin and 28% amoxicillin/clavulanic acid in 2015* (*p* = 0.02))*.*

## Discussion

This study is the first that focused on the trend of antibiotic prescriptions in time in a secondary paediatric care setting in Northern Europe. Although no significant trend in total DOT/100PD could be observed on the paediatric and neonatology ward, trends regarding specific antibiotic classes, route, and duration were observed. This setting differs from a tertiary setting because of the absence of haemato-oncology and intensive care patients: a population with a high antibiotic consumption. However, since antibiotics are frequently prescribed in all fields of paediatrics, it is important to investigate all possibilities for reducing antibiotic use in the light of antimicrobial stewardship.

The average DOT/100PD for the paediatric ward in our study was between 35 and 45. Recent data from smaller and larger American community hospitals showed the median DOT/100PD over 2011–2013 for paediatric wards including surgery to be 49, somewhat higher compared to our figures [23]. Ideally, figures should be compared with secondary paediatric wards from an identical setting enabling doctors to identify differences in and rationales for their prescription behaviour. Other studies reporting a comparable setting are not yet available. A Canadian children’s hospital study showed DOT/100PD to reduce from ~94 to 76 in about the same time period (2011–2013) [3]. Average DOT/100PD in American freestanding children’ hospitals ranged from ~77 in 2004 to ~65 in 2012 [10]. Another American study analysing data from university hospitals from 2002 to 2007 showed DOT/100PD to be 54–56; no trend in time was observed, but this study was performed before the formal ASP were initiated [20]. In our study, the secondary care setting and the restricted antibiotic use in the Netherlands in general might be the main factors explaining the lower use of antimicrobial agents and the absence of a trend in total DOT/100PD compared to the other American studies, including the community hospital study. A recent study reported the outcome of an ASP at tertiary general paediatric wards (excluding surgery) in Germany, calculating a DOT/100 PD of 48 before intervention and 43 after intervention [14]. This setting can probably be best compared to a secondary care setting. The ARPEC studies showed point prevalence (PP) of antibiotic use in general medical paediatric and neonatal wards of 39 and 12.3%, respectively. These are merged data from different hospitals worldwide (both tertiary and secondary); the UK data show a PP for general secondary and tertiary wards of 36.4 and 37.5%. This metric can also not be fully compared because of the different denominator. However, the point prevalence is an easier metric since it can be calculated without the need for hospital information technology services, but may not portray the magnitude of antibiotic use over time [8, 24].

The year 2015 had a somewhat lower, although not statistically significant, paediatric DOT/100PD compared to the other years. Since 2014, antibiotic case rounds and teaching have been installed in this hospital to make the staff and junior doctors more aware of the possible consequences of antimicrobial treatment. During the case rounds, all infectious patients are reviewed and indications and choices for antimicrobial therapy are discussed. Additionally, antibiotic prescriptions are discussed at the morning report. Whether this will lead to a sustained trend or is the result of temporal changes in case mix should be analysed in the future. The neonatology department showed a strongly fluctuating DOT/100PD with a non-significant increase from 24 to 34; this might be explained by case mix (e.g. higher patient turnover in 2015).

Third-generation cephalosporin consumption and the intravenous route for the paediatric ward were significantly lower over the years; this was shown both in the global DOT study and the supporting UTI/LRTI study.

Unfortunately, admission figures to calculate the patient days from before 2010 could not be reliably obtained.

This study identified that the total duration of antibiotic therapy in patients with LRTI significantly decreased. In addition, intravenous antibiotic treatment decreased in both patients with LRTI and UTI. A recent review emphasised the importance of switching to oral agents as soon as clinical improvement has occurred [15]. The observed reductions in antibiotic use in these groups could be attributable to increased awareness of antimicrobial resistance and a selection of interventions in the context of antimicrobial stewardship. Regular feedback and new protocol development aided in the reduced use of broad-spectrum cephalosporins and penicillins. In 2014, a new local UTI protocol was implemented with emphasis on an early iv-oral switch and empirical treatment with intravenous or oral amoxicillin/clavulanic acid in the absence of risk factors such as prior culture results with resistant pathogens. The amoxicillin/clavulanic acid resistance in paediatric *E. coli* isolates was still low (< 20%) in this hospital, but might change in the future. Additionally, a national childhood community-acquired pneumonia (CAP) protocol was published, emphasising the limited value of additional investigations and the shorter duration of antibiotics (5 days) needed for non-complicated LRTI. In our setting, regular feedback and local and national protocols have definitely created awareness amongst doctors regarding the preferred spectrum, route, and duration of antibiotics for LRTI. Moreover, significantly less diagnostic tests were requested. In other studies, identical results were observed [18, 22]. After implementing stewardship practices, cephalosporin and amoxicillin/clavulanic acid use for CAP could be restricted and replaced by amoxicillin.

Strikingly, the percentage of patients who were administered any antibiotic for LRTI did not decrease in our study, but the number of additional investigations for LRTI decreased significantly. Education about the aetiology of LRTI in infants could possibly further reduce the percentage of infants receiving antibiotics for a LRTI, which are mostly viral in nature.

The strength and originality of this study is that it focuses on both total and detailed aspects of antibiotic prescriptions. Apart from analysing inpatient antibiotic use by DOT, this study also evaluated the in- and outpatient antibiotic use for one infectious disease episode, which together reflect the full antibiotic prescription. Both analyses showed a decrease in third-generation cephalosporin use over the years. Although the specific diseases sub study only compared 2 years, the outcomes are supported by the DOT study and the interventions in the context of ASP. This study was restricted to one centre; consequently, implementations and outcomes might not be extrapolated to other clinical centres. Ideally DOT’s should be calculated (semi)-automatically by hospital software programs. This allows for comparing DOT’s easily between time periods and hospitals. However, using DOT’s in paediatrics also has some limitations. DOT’s include the assumption that antibiotic dosing is appropriate, and the quality of the prescriptions cannot be assessed, e.g. broad-spectrum monotherapy leading to fewer DOT’s compared to narrow-spectrum combination therapy.

Future research should be aimed towards evaluating comparable care units with comparable resistance rates and understanding differences in antibiotic prescribing and barriers to rational prescribing. This will aid in developing national antimicrobial stewardship priorities and allow for more selective antimicrobial use.

Concluding, in this secondary setting, a significant decrease in overall inpatient cephalosporin use and cephalosporin use for UTI and LRTI was identified. No trend in total DOT/100PD could be observed for both the neonatal and paediatric wards. However, the total intravenous administration was shortened in time. The total treatment duration and proportion of broad-spectrum beta-lactam antibiotics significantly decreased for LRTI. Increased awareness of antimicrobial resistance and a selection of interventions in the context of antimicrobial stewardship could explain this decrease in antibiotic use. This decrease in antibiotic use is feasible and important in non-tertiary paediatric wards and outpatient clinics.

## Electronic supplementary material

ESM 1 (DOCX 159 kb)

ESM 2 (DOCX 79 kb)

ESM 3 (DOCX 80.9 kb)

## Abbreviations

ASP: Antimicrobial stewardship programmes
ATC: Anatomical therapeutic chemical
CAP: Community-acquired pneumonia
DOT/100 PD: Days of therapy per 100 patient-days
DDD: Defined daily dose
ENT: Ear nose throat
IDSA: Infectious Diseases Society of America
LRTI: Lower respiratory tract infection
PP: Point prevalence
TMP/SMX: Trimethoprim/sulfamethoxazole
UTI: Urinary tract infection
WHO: World Health Organization
