# Supplementary material for: Trends in paediatric inpatient antibiotic therapy in a secondary care setting
Source: Eur J Pediatr. 2018 Jun 8;177(8):1271–8. doi: 10.1007/s00431-018-3185-z (PMC6061058; doi:10.1007/s00431-018-3185-z)
Supplement: Supplementary file 1 — (DOCX 159 kb) [file 431_2018_3185_MOESM1_ESM.docx]

**Supplementary figures (PDF files figures seperately sent)**

**Figure 1a**

Ratio of oral and intravenous DOT/100PD neonatal ward. Intravenous DOT R^2^=0.40; p=0.18

**Figure 1b**

Ratio of oral and intravenous DOT/100PD paediatric ward. Intravenous DOT R^2^=0.63; p=0.06.
