# Supplementary material for: Trends in paediatric inpatient antibiotic therapy in a secondary care setting
Source: Eur J Pediatr. 2018 Jun 8;177(8):1271–8. doi: 10.1007/s00431-018-3185-z (PMC6061058; doi:10.1007/s00431-018-3185-z)
Supplement: Supplementary file 2 — (DOCX 79 kb) [file 431_2018_3185_MOESM2_ESM.docx]

**Supplementary table 1**

**Baseline characteristics of patients with urinary tract infection**

| Characteristics (unit) | 2008 (n=27) | 2015 (n=11) | p-value* |
| --- | --- | --- | --- |
| Median age (years) (IQR) | 0.9 (0.2-2.1) | 0.4 (0.2-0.9) | 0.46 |
| Gender – No. male (%) | 19 (70.4) | 3 (27.3) | 0.03 |
| Fever – No. (%) | 24 (88.9) | 8 (72.7) | 0.33 |
| Anatomic urinary tract abnormality – No. (%) | 7 (25.9) | 3 (27.3) | 1.00 |
| Median CRP (IQR) (mg/l) | 55.0 (15.0-97.0) | 120.5 (12.0-197.0) | 0.21 |
| Median WBC (IQR) (10^9^/l) | 19.7 (11.1-24.0) | 15.6 (11.3-22.1) | 0.98 |
| Blood culture – No. (%) | 16 (59.3) | 4 (36.4) | 0.20 |
| No growth  Positive culture  *E. coli* | 13 (48.1)  3 (18.8)  3 (18.8) | 4 (36.4)  0 (0)  - |  |
| Urine culture – No. (%) | 26 (96.3) | 11 (100) |  |
| No growth  Positive culture  *E. coli*  Res A/C (%)  Res CEF3 (%)  Res TMP/SMX (%)  *Klebsiella spp.*  Res A/C (%)  Res CEF3 (%)  Res TMP/SMX (%)  *Citrobacter spp.*  Res A/C (%)  Res CEF3 (%)  Res TMP/SMX (%) | 4 (14.8)  22 (84.6)  19 (86.4)  1 (5.3)  1 (5.3)  7 (31.8)  1 (4.7)  0 (0)  0 (0)  0 (0)  1 (3.7)  1 (100)  0 (0)  0 (0) | 2 (18.2)  9 (81.8)  8 (72.7)  0 (0)  0 (0)  0 (0)  1 (9.1)  0 (0)  0 (0)  1 (9.1)  -  - |  |

*Mann-Whitney U test or Fisher’s exact test

A/C: amoxicillin/clavulanic acid

CEF3: third generation cephalosporin

TMP/SMX: trimethoprim/sulfamethoxazole

Res: resistance
