# Supplementary material for: Trends in paediatric inpatient antibiotic therapy in a secondary care setting
Source: Eur J Pediatr. 2018 Jun 8;177(8):1271–8. doi: 10.1007/s00431-018-3185-z (PMC6061058; doi:10.1007/s00431-018-3185-z)
Supplement: Supplementary file 3 — (DOCX 80.9 kb) [file 431_2018_3185_MOESM3_ESM.docx]

**Supplementary table 2.**

**Baseline characteristics of patients with lower respiratory tract infection**

| Characteristics (unit) | 2008 (n=73) | 2015 (n=97) | p-value* |
| --- | --- | --- | --- |
| Median age (years) (IQR) | 1.8 (0.7-3.9) | 1.6 (0.6-3.3) | 0.51 |
| Gender – No. male (%) | 42 (57.5) | 40 (41.2) | 0.04 |
| Fever - No. (%) | 56 (76.7) | 77 (79.4) | 0.68 |
| Comorbidities – No. (%)  *Premature birth*  *Syndrome/retardation*  *Immunodeficiency* | 15 (20.5)  8  7  0 | 26 (26.8)  11  14  1 | 0.35 |
| CRP - No. (%)  Median CRP (mg/l) (IQR) | 66 (90.4%)  46.0 (18.0-108.0) | 66 (68.0%)  25.5 (11.0-56.0) | 0.00  0.02 |
| WBC - No.(%)  Median WBC (10^9^/l) (IQR) | 61 (83.6%)  16.8 (11.5-21.2) | 63 (64.9%)  10.8 (8.7-15.7) | 0.01  0.00 |
| Chest X-ray - No. (%)  *CXR abnormal*  *CXR unclear*  *CXR normal* | 67 (91.8%)  42  17  8 | 61 (62.9%)  44  8  9 | 0.00 |
| Blood culture – No. (%) | 26 (35.6) | 18 (18.6) | 0.01 |
| No growth  Positive culture  *S. pneumoniae* | 26 (35.6)  0 (0.0)  - | 17 (17.5)  1 (1.0)  1 (1.0) |  |
| Sputum culture - No. (%) | 3 (4.1) | 3 (3.1) |  |
| No growth  Positive culture  *H. influenzae* | 1 (1.4)  2 (2.7)  2 (2.7) | 1 (1.0)  2 (2.1)  2 (2.1) |  |

* Chi-square test or Mann-Whitney-U-test
